# Boruta algorithm–guided antibiotic selection in antibiotic-loaded bone cement for diabetic foot ulcers: microbiota and susceptibility analysis

**DOI:** 10.3389/fphar.2025.1677198

**Published:** 2025-09-23

**Authors:** Yi Zhang, Xingyu Sun, Maochun Wu, Xin Tang

**Affiliations:** ^1^ Department of Orthopedics, The First Affiliated Hospital of Dalian Medical University, Dalian, Liaoning, China; ^2^ Department of Hand and Foot Surgery, Zibo Central Hospital, Zibo, Shandong, China; ^3^ School of Mechanical and Electrical Engineering, Jining University, Jining, China; ^4^ Jining polytechnic, Jining, China

**Keywords:** diabetic foot ulcer, antibiotic-loaded bone cement, bacterial microbiota, antibiotic sensitivity analysis, feature selection

## Abstract

**Background:**

Diabetic foot is one of the serious complications of diabetes mellitus, and diabetic foot ulcer (DFU) infection is often fatal to patients. As a relatively new method, antibiotic bone cement treatment of diabetic foot is beneficial to many patients with diabetic foot infection (DFI). The aim of this study is to analyze the composition of DFU wound microbiota and to screen antibiotics with better efficacy to add to bone cement for the treatment of DFI.

**Methods:**

We collected the exudates from DFU wounds of patients for bacterial culture and drug susceptibility test, and classified and analyzed the composition of bacterial microbiota. For drug susceptibility test results, we first screened the antibiotics by using the Boruta algorithm prediction model and then performed sensitivity analysis based on age and gender factors.

**Results:**

According to this study, age was a significant factor in the cumulative sensitivity of bacteria to antibiotics added with bone cement. In the bacterial microbiota of DFU wounds, Gram-positive bacteria accounted for a relatively high proportion, and the main species was *Staphylococcus aureus*. Based on the analysis, the better choices of antibiotics were Gentamicin (GEN) and Tobramycin (TOB) for Gram-negative bacteria and Moxifloxacin (MFX), Ampicillin (AMP) and Quinupristin-dalfopristin (QDA) for Gram-positive bacteria.

**Conclusion:**

In the treatment of DFI, Gram-positive bacteria infection is more common in clinical practice, especially anti-infective treatment against *Staphylococcus aureus* needs to be paid attention to. Based on our findings, it is advised that the DFU population who requires bone cement antibiotic treatment be treated according to age, with GEN and TOB being recommended for Gram-negative bacteria and MFX, AMP, and QDA for Gram-positive bacteria.

## 1 Introduction

The prevalence of diabetes has rapidly expanded worldwide as a result of modern development, and complications such as diabetic foot have also developed in tandem (Pérez-Panero et al., 2019). Diabetic foot ulcer (DFU) is the most common chronic wound. The global prevalence of DFU is 6.3% (Zhang et al., 2017), and DFU is the main cause of amputation in patients with diabetes (Madanchi et al., 2013). As many as 15% of diabetes patients are affected by diabetes foot infections (DFI). The annual mortality rate for patients with diabetic foot infections is as high as 12%, while the mortality rate for those who undergo amputation is even as high as 24% (Mendame Ehya et al., 2021; Schaper et al., 2020). One of the main causes of hospitalization and toe amputation for diabetic foot patients is DFI (Saltoglu et al., 2015). Diabetic foot is mostly caused by diabetic peripheral artery disease and neuropathy. Peripheral neuropathy causes limbs to lose their protective feeling, making it difficult to detect early ulcers in time. This might result in ulcer infection as the disease progresses. The infected ulcer wound cannot heal quickly due to the complex condition, which has a major Gram-negative impact on patients' quality of life and adds to the burden on families and society (Jones et al., 2018).

Numerous related studies are already available to examine the features of the microbiota in DFU wounds. At the wound site, DFI is primarily colonized by a range of microorganisms, primarily bacteria. The growth and reproduction of these microorganisms causes tissue damage and inflammatory responses. Peripheral vascular disorders are common in DFI patients, making it challenging to eradicate germs and manage infection. Additionally, the infection is easily contagious. Diabetic foot osteomyelitis (DFO) may result in extreme situations (Waaijman et al., 2014). Effective anti-infection therapy is therefore the cornerstone of DFI treatment, and identifying the distribution of harmful bacteria and their medication resistance serves as a crucial foundation for developing treatment plans. Due to their inability to accurately assess DFI pathogens, many clinicians are only able to use antibiotics empirically in the early stages. This can easily result in poor treatment outcomes and the emergence of drug-resistant bacteria, making it more difficult to choose effective antibiotics in the future (Miyan et al., 2017).

Even while scientists have made great strides in understanding how DFU wounds occur and how to treat them, there are still many obstacles to overcome before diabetic foot may be fully cured. Evaluation of the foot, antibacterial medication treatment, debridement and dressing change, wound dressing, Gram-negative pressure wound treatment, blood supply reconstruction, and patient and family education are all common clinical treatment modalities for DFI wounds (Schaper et al., 2020; Lim et al., 2017; Jones and Harding, 2015). Nevertheless, DFI has a poor therapeutic impact, and the healing process is lengthy. Consequently, hospital stays are frequently longer and medical costs are rising. The application of new technologies and methods has accelerated the efficacy of drug treatment. The latest developments in nanotechnology have provided a variety of approaches for studying and addressing various medical issues and diseases at the nanoscale. Nanoparticles can help diagnose and treat diseases by directly delivering drugs to target cells, which will significantly improve the treatment effect of patients and reduce the time, energy and funds required for diagnosis and treatment of diseases. It can also reduce the side effects of the treatment and provide more precise and effective treatment. Bone cement, as a nano-scale carrier, can carry antibiotics and release them continuously and slowly, allowing for local administration to treat infected wounds on diabetic feet (Raghunath et al., 2025; Bukke et al., 2024; Pattnaik et al., 2023). The course of systemic antibiotic treatment for DFI is generally no less than 6 weeks (Pattnaik et al., 2023), and long-term use of antibiotics will lead to adverse reactions and bacterial resistance, and cause additional side effects on some organs (Zeun et al., 2016). As a kind of nano-biological material, antibiotics are stored in the bone cement matrix as a carrier and released slowly. The high concentration of the drug is a necessary condition for exerting its antibacterial effect. The release of antibiotics from the bone cement matrix depends on the surface area and follows the principle of diffusion. The diffusion of the drugs is closely related to the absorption of water. The release of these drugs is proportional to the water absorption characteristics of the bone cement, and the release time also needs to be taken into account (Kuhn, 2013). Antibiotic bone cement can release a large amount of antibiotics locally, thereby reducing the risk of systemic toxicity and successfully preventing and treating infections. According to recent research, treating DFI wounds with bone cement that contains antibiotics has also produced impressive therapeutic results. The effects of vacuum sealing drainage (VSD) and antimicrobial bone cement in the management of DFU wounds were examined in a retrospective case study. According to the findings, antibacterial bone cement has a greater impact than VSD treatment in promoting wound healing in Wagner 3-4 diabetic patients by efficiently reducing infection and speeding up the healing cycle (Sun et al., 2022). In recent years, some scholars have utilized nanotechnology to develop a drug delivery technology using nano-cube crystals for the treatment of rheumatoid arthritis (Chandrakala et al., 2025). Some researchers have also used β-cyclodextrins conjugated nanoparticles to deliver drugs for the treatment of cancer (Yadav et al., 2025). These technologies have expanded some treatment methods for difficult-to-treat diseases and have also promoted the development of pharmacotherapy.

Selecting antibiotics to enhance the curative impact of bone cement can be difficult, despite the fact that antibiotic bone cement works well for DFI. No academics have done any pertinent research as of yet. The experience of antibiotic-loaded bone cement in treating periprosthetic infections is the basis for numerous clinical investigations, the majority of which combine gentamicin and vancomycin (Li et al., 2022; Bitsch et al., 2015). When antibiotics are injected into bone cement and applied locally to diabetic foot ulcers, it can quickly result in drug resistance and poor outcomes. Thus, in order to improve the effectiveness of antibiotic-loaded bone cement in treating DFU wounds, we further examine the microbiota of DFU wounds through this study and evaluate the data using a machine learning algorithm to further acquire effective antibiotic recommendations.

Therefore, we collected specimens and analyzed the characteristics of the bacterial community in diabetic foot diseases, then conducted antibiotic susceptibility screening based on clinical case data to minimize patient pain and treatment burden, and to guide the combination of antibiotics with bone cement therapy in diabetic patients. This study aimed to collect and analyze the characteristics of the bacterial flora in diabetic foot wounds, and based on the types of bacteria and the relevant drug sensitivity results, determine the types of antibiotics with high sensitivity for treating diabetic foot ulcers using the Boruta algorithm.

## 2 Materials and methods

### 2.1 Dataset collection and preprocessing

Participants in this study were Zibo Central Hospital patients with type 2 diabetes receiving treatment for diabetic foot. All information was taken from the hospital medical record system between January 2020 and December 2024. All patients had surgery using bone cement containing antibiotics, and prior to surgery, a sample of the diabetic foot’s ulcer area was taken for further bacterial culture and medication sensitivity testing. We gathered data regarding these patients’ bacterial cultures, drug sensitivity tests, age, and sex.

### 2.2 Inclusion criteria

i. individuals with type 2 diabetes who met the specific criteria for diagnosis (fasting plasma glucose≥7.0 mmol/L, 2-h glucose≥11.1 mmol/L, or glycosylated hemoglobin≥6.5%);
ii. problems related to diabetic foot infection; The severity of diabetic foot was assessed according to Wagner grading, and the included patients were evaluated as Wagner grades 3–4. The bacterial culture of the wound secretion of sugar foot was positive.
iii. verbal agreement from the patient.

### 2.3 Exclusion criteria

Type 1 diabetes, gestational diabetes, thromboangiitis obliterans, tumors, no infection diabetic foot patients and inadequate clinical data.

### 2.4 Ethics approval and informed consent

The study was approved by the medical ethics committee of the ZiBo Central Hospital (Approval number: 2024068) on 04/23/2024, which was consistent with medical ethics. The study was a retrospective study and the data of included patients were anonymous. The study was informed consent was obtained from all subjects and/or their legal guardian(s). During the research, the World Medical Association (WMA) Declaration of HELSINKI and/or the World Psychiatric Association HAWAII Declaration of Good Clinical Practice rules were complied with.

### 2.5 Bacteria cultivation, isolation identification and drug susceptibility test

Before or during treatment, first, disinfect the skin around the wound with alcohol to prevent contamination, the secretions from infected wounds of diabetic foot were dipped in sterile cotton swabs, placed in sterile test tubes, then it was placed into the transferer, and immediately sent for examination in 2 h. Microscan WalkAway 96 plus (Beckman, United States) or VITEK 2 Compact (Meyer, France) automatic microbial analysis system is used for strain identification and antimicrobial susceptibility testing. The results of antimicrobial susceptibility testing were determined in accordance with the latest CLSI criteria (as applicable each year). The minimum inhibitory concentrations (MICs) were used to detect methicillin-resistant *S. aureus*. Quality control strains include *S. aureus* (ATCC 25923), *E. coli* (ATCC25922), and *P. aeruginosa* (ATCC27853) (National Health Commission Clinical Laboratory Center). The cultivated bacteria have undergone additional classification and analysis (Please refer to <2.8 Analysis process for this study > for the relevant procedures). We do not routinely culture anaerobic bacteria and special flora. Further culture and identification are only conducted under highly suspicious or special circumstances. This limits the comprehensive identification of bacteria. Test for drug sensitivity, for Gram-positive bacteria, we utilized 16 different types of antibiotics, and for Gram-negative bacteria, we used 20 different types (Table 1). We compared the antibiotics that can be added to bone cement based on the Consensus of Periprosthetic Infection and the relevant study conducted by German researcher Wahlig (Parvizi et al., 2013; Wahlig, 1987).

**TABLE 1:** The type of antimicrobial used in the bacterial susceptibility test.

The type of bacteria	The type of antimicrobial	The number of types
Germ-positive bacteria	Penicillin (PEN); Oxacillin (OXA); Ampicillin (AMP); Vancomycin (VAN); Linezolid (LNZ); Levofloxacin (LVF); Sulfamethoxazole (SXT); Gentamicin (GEN); Clindamycin (CLI); Erythromycin (ERY); Rifampicin (Rif); Tetracycline (TCY); Tigecycline (TIG); Quinupristin-dalfopristin (QDA); Moxifloxacin (MFX); Ciprofloxacin (CIP)	16
Germ-negative bacteria	Piperacillin/tazobactam (TZP); Cefoperazone Sodium/sulbactam Sodium (CLS); Meropenem (MEM); Imipenem (IPM); Ceftazidime (CAZ); Aztreonam (AZT); Amikacin (AMK); Levofloxacin (LVX); Polymyxin B(POLB); Tigecycline (TIG); Cefepime (FEP); Cefotaxime (CEF); Cefuroxime (CXM); Ciprofloxacin (CIP); Piperacillin (PIP); Ampicillin (AMP); Minocycline (MIN); Doxycycline (DOX); Tobramycin (TOB); Gentamicin (GEN)	20

### 2.6 Data analysis methods

#### 2.6.1 Boruta algorithm

In general, the prediction outcomes are significantly impacted by feature selection. A feature selection technique called the Boruta algorithm was created to find every pertinent feature in a dataset, especially when supervised learning is involved. As an extension of the Random Forest method, it was put forth by Breiman (2001) with the goal of differentiating between features that are genuinely significant and those that are noise or redundant. Unlike traditional feature selection method, Boruta adopts an “all-relevant” approach, ensuring that no significant feature is overlooked. All input features’ Z-scores are calculated by the method, and the distribution uses the Z-score metrics to identify the key variables and shadow features of the predictors. The methods used are listed below.

For each original feature $X_{i}$ , create a shuffled version $X_{i}^{shadow}$ to serve as a decoy. Shadow features act as a baseline for randomness. For each original feature, compare its importance to the maximum importance of all shadow features. If the feature’s importance is higher, it gets a “hit”. Compute the mean and standard deviation of shadow feature importance. For each original feature, calculate a Z-score.

$$Z_{i}=\frac{Importance\left(X_{i}\right)-Mean\left(Shadow\;Importance\right)}{SD\left(Shadow\;Importance\right)}$$

*Z*_*i*_ is the Z-score for the original feature *X*_*i*_, measuring how many standard deviations its importance is above or below the mean importance of shadow features. Importance (*X*_*i*_) is the importance score of the original feature *X*_*i*_. Mean (Shadow Importance) is the average importance score of all shadow features. SD (Shadow Importance) is the standard deviation of shadow feature importance. In this study, drugs related to age and gender were screened by random forest Boruta algorithm. Screening the correlation between different drugs and patients’ age and gender, so as to screen out the susceptibility of different ages and genders to different drugs.

After obtaining the drug sensitivity results, we first choose the drugs recommended in the fever treatment or related guidelines for DFU infections. At the same time, we select the drugs with lower MIC values based on the drug sensitivity results (This value was provided by our microbiology laboratory. It is classified based on the MIC values of different bacteria and antibiotics as follows: intermediate (I); resistant (R); sensitive (S)). However, in empirical treatment, we will take into account the drug resistance trend of our hospital and avoid choosing drugs with a higher resistance rate. For example, when the risk of bacterial infection is high, we avoid using levofloxacin (The drug resistance rate is around 60%). After collecting the drug sensitivity data, the relevant results were obtained by applying the Boruta algorithm.

#### 2.6.2 Feature selection and sensitivity analysis

Feature selection is the process of selecting a subset of relevant features (variables, predictors) for use in model construction. It's a crucial step in machine learning and data analysis that can improve model performance, reduce overfitting, and enhance interpretability.

Risk model sensitivity analysis enables the identification of predominant exposure factors, facilitating data-driven prioritization of risk control measures. Sensitivity analysis methods have found widespread application across diverse disciplines, ranging from complex engineering systems and economics to physics, social sciences, and medical decision-making. In this study, the drug sensitivity was analyzed according to the age and gender of the patients. In this paper, sensitivity analysis and data visualization was generated using R 4.3.0.

The use of antibiotics is indeed closely related to age. There are significant differences among different age groups in terms of the types of infections, the types of antibiotics commonly used, the dosage of medication, and the risks involved. The use of antibiotics varies by age, and the main influencing factors include the following points: i) Immune system maturity: The immune systems of infants and the elderly are relatively weak or incomplete, making them more susceptible to infections, but also more prone to being affected by the side effects of antibiotics. ii) Common pathogen types: The common pathogens vary across different age groups (for instance, viral infections are more common in children, but the bacterial pathogens also differ from those in adults). iii) Organ function development and decline: The liver and kidneys are the main organs for metabolizing and excreting antibiotics. The liver and kidney functions of infants and young children are not yet fully developed, while those of the elderly may decline, which directly affects the dosage and selection of drugs. iv) Pharmacokinetic differences: The processes of drug absorption, distribution, metabolism and excretion in the body vary with age. Therefore, when conducting the sensitivity analysis, we selected two factors: age and gender.

### 2.7 Analysis process for this study

**FIGURE 1:**
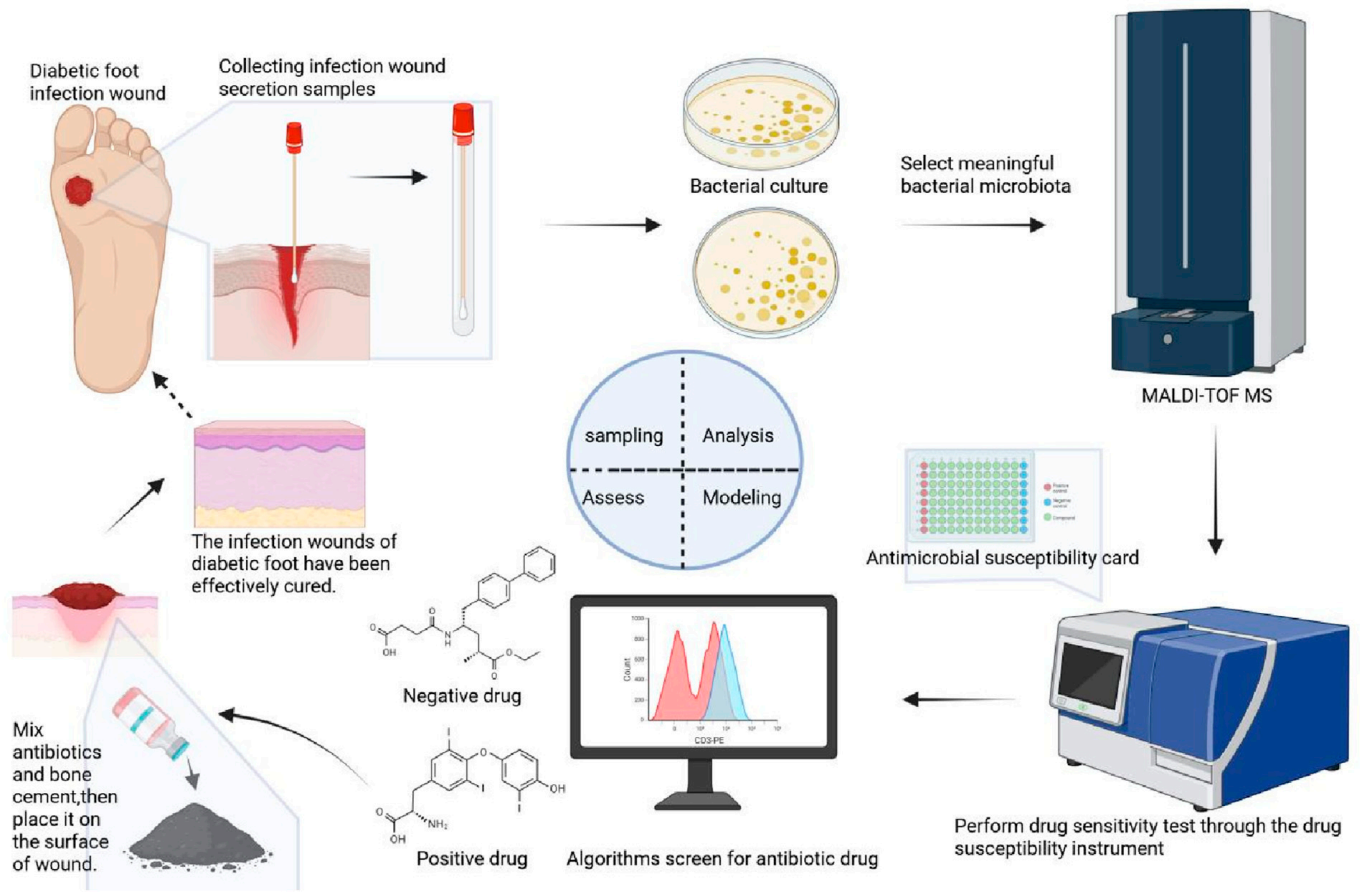
The overall framework of the antibiotic screening process.

The screening process is shown in Figure 1. As shown in Figure 1, the approach was created in four basic steps. First, infected wound secretions were wiped with a sterile cotton swab and sampled, the collected swabs were then inoculated on the AGAR (Columbia Blood Agar Plate) surface. After 24 h of incubation, significant colonies were selected for species identification by Matrix Assisted Laser Desorption Ionization Time of Flight Mass Spectrometry (MALDI-TOF MS). Then, reasonable drug susceptibility cards were selected for drug susceptibility testing (The susceptibility of the drug was determined according to the CLSI standard using the broth microdilution method.), then sensitive drugs can be screened. Thirdly, through the feature extraction algorithm, the sensitivity characteristics related to the patient’s bacterial drug susceptibility test are screened and analyzed, and the preferred bacterial sensitive drugs are obtained. Finally, the types of drugs that can be added with bone cement are further screened out.

**FIGURE 2:**
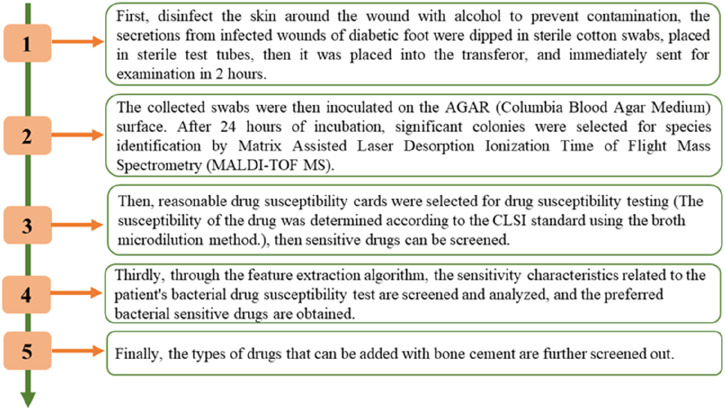
The detailed diagram of the methodology.

The diagram of the methodology is shown in Figure 2. As shown in Figure 2, detailed diagram of the methodology have added along with various time points.

## 3 Results

### 3.1 General characteristics of research subjects

We extracted a large number of test data of patients with DFI from the database of Zibo Central Hospital. After finally passing the screening, the test data of 102 patients were included in the study, of which 75 were male and 27 were female. Ages range from 16 to 90. Of these, 36 patients experienced recurrence following surgery, while 66 patients were cured by antibiotic-loaded bone cement treatment. (Table 2).

**TABLE 2:** Basic characteristics of the included patients.

Item	Proportion of characteristic
Sex (man/woman)	75:27 (74%:26%)
Age range (year)	16–90
The proportion of Mono-bacterial and multiple bacteria infection	83:19 (81%:19%)
The proportion of germ-positive bacteria and germ-negative bacteria	75:45 (63%:37%)
Proportion of surgical effect (cure: palindromia)	66:36 (65%:35%)

### 3.2 Distribution of pathogenic bacteria

**FIGURE 3:**
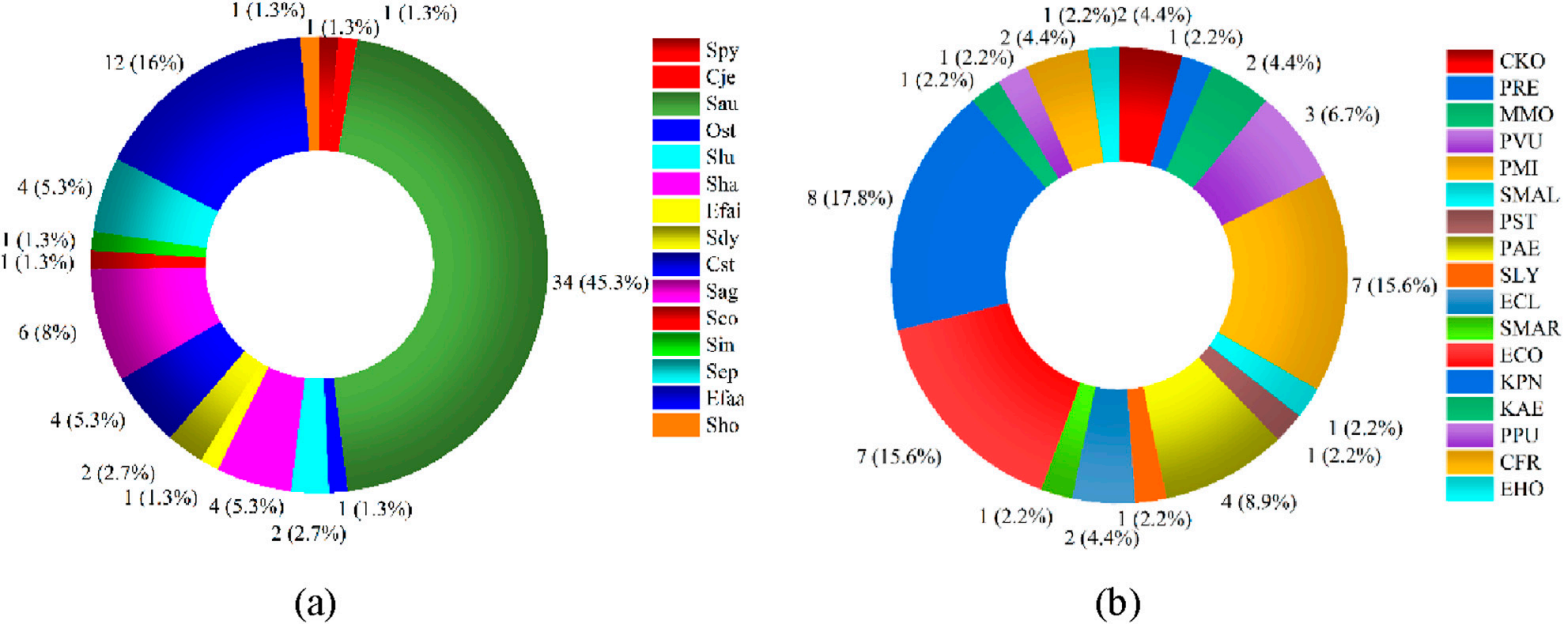
Distribution of germ-positive and Gram-negative bacteria. **(a)** Germ-positive bacteria **(b)** Germ-negative bateria.

Among 102 patients, 83 cases were infected by single bacteria and 19 cases were infected by multiple bacteria. Among them, there were 75 g-positive bacteria and 15 strains, among which *Staphylococcus aureus* was the most, accounting for 34.46%, and *Enterococcus faecalis* was 12.16% (Figure 3a). There were 45 cases of Gram-negative bacteria and 17 species of bacteria, among which *Klebsiella pneumoniae* accounted for 8.18%, *Escherichia coli* and *Proteus mirabilis* accounted for 7.16% (Figure 3b). Distribution of germ Gram-positive and Gram-negative bacteria can be seen Figures 3a,b. The proportion and number of germ Gram-positive and Gram-negative bacteria can be seen Table 3.

**TABLE 3:** The proportion of germ-positive and Gram-negative bacteria.

The type of bacteria	Bacteria	The number of bacteria	The proportion (%)
Germ-positive bacteria	Spy	1	1.33
	Cje	1	1.33
	Sau	34	45.33
	Ost	1	1.33
	Slu	2	2.67
	Sha	4	5.33
	Efai	1	1.33
	Sdy	2	2.67
	Cst	4	5.33
	Sag	6	8.0
	Sco	1	1.33
	Sin	1	1.33
	Sep	4	5.33
	Efaa	12	16.0
	Sho	1	1.33
Germ-negative bacteria	CKO	2	4.44
	PRE	1	2.22
	MMO	2	4.44
	PVU	3	6.67
	PMI	7	15.56
	SMAL	1	2.22
	PST	1	2.22
	PAE	4	8.89
	SLY	1	2.22
	ECL	2	4.44
	SMAR	1	2.22
	ECO	7	15.56
	KPN	8	17.78
	KAE	1	2.22
	PPU	1	2.22
	CFR	2	4.44
	EHO	1	2.22

### 3.3 Analysis of drug susceptibility results

**FIGURE 4:**
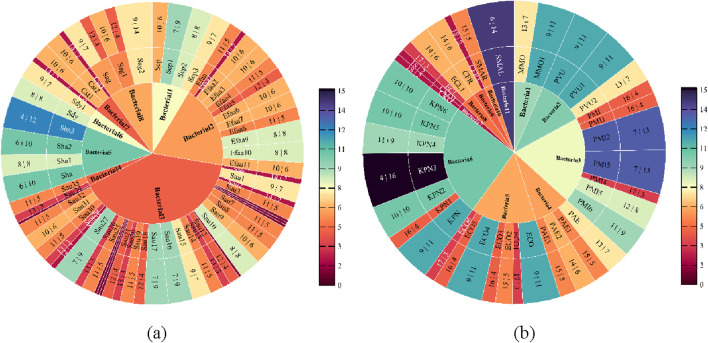
Drug sensitivity analysis of Gram-positive and Gram-negative bacteria. **(a)** Sensitivity analysis of Gram-positive bacteria **(b)** Sensitivity analysis of Gram-negative bacteria.

We calculated statistics on the drug sensitivity of the primary Gram-positive and Gram-negative microorganisms. The ring chart’s outermost numbers indicate the number of medications that are responsive to and insensitive to various drugs, respectively. There are eight major types of Gram-positive bacteria (Figure 4a) and ten types of Gram-negative bacteria (Figure 4b). The bacteria’s susceptibility to medications decreases with increasing blueness. Additionally, Figures 4a,b show that Gram-positive bacteria are more drug-sensitive than Gram-negative bacteria.

### 3.4 Feature importance analysis via Boruta algorithm

**FIGURE 5:**
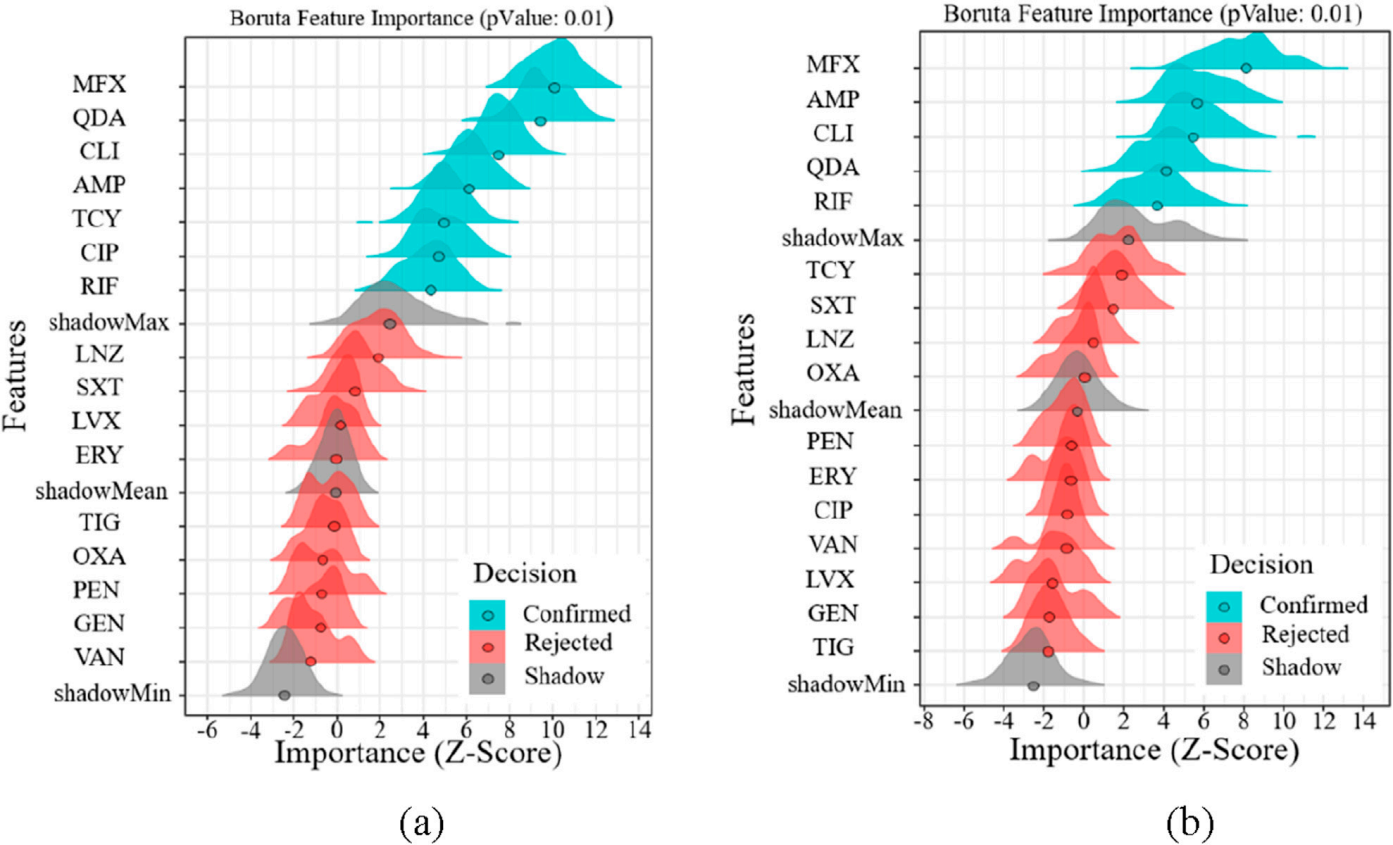
Feature importance analysis of Gram-positive bacteria. **(a)** Feature importance analysis of gender **(b)** Feature importance analysis of age.

In order to filter out the linked pharmaceuticals that have the most relevant influence on age and gender and to further study the drug susceptibility, the susceptibility of various drugs to both Gram-positive and Gram-negative bacteria was chosen using age and gender as key variables. We started by examining the medication susceptibility of the microorganisms that tested positive. The most gender-related medications include MFX, QDA, CLI, AMP, TCY, CIP, and RIF, as shown in Figure 5a. The medications that are most associated with aging include MFX, AMP, CLI, QDA, and RIF, as shown in Figure 5b. Figure 5 illustrates how gender exhibits more drug susceptibility to Gram-positive bacteria and how the Boruta algorithm is important in filtering out additional medication types. MFX, QDA, CLI, AMP, and RIF are among the medications that are susceptible to Gram-positive bacteria according to age and gender.

**FIGURE 6:**
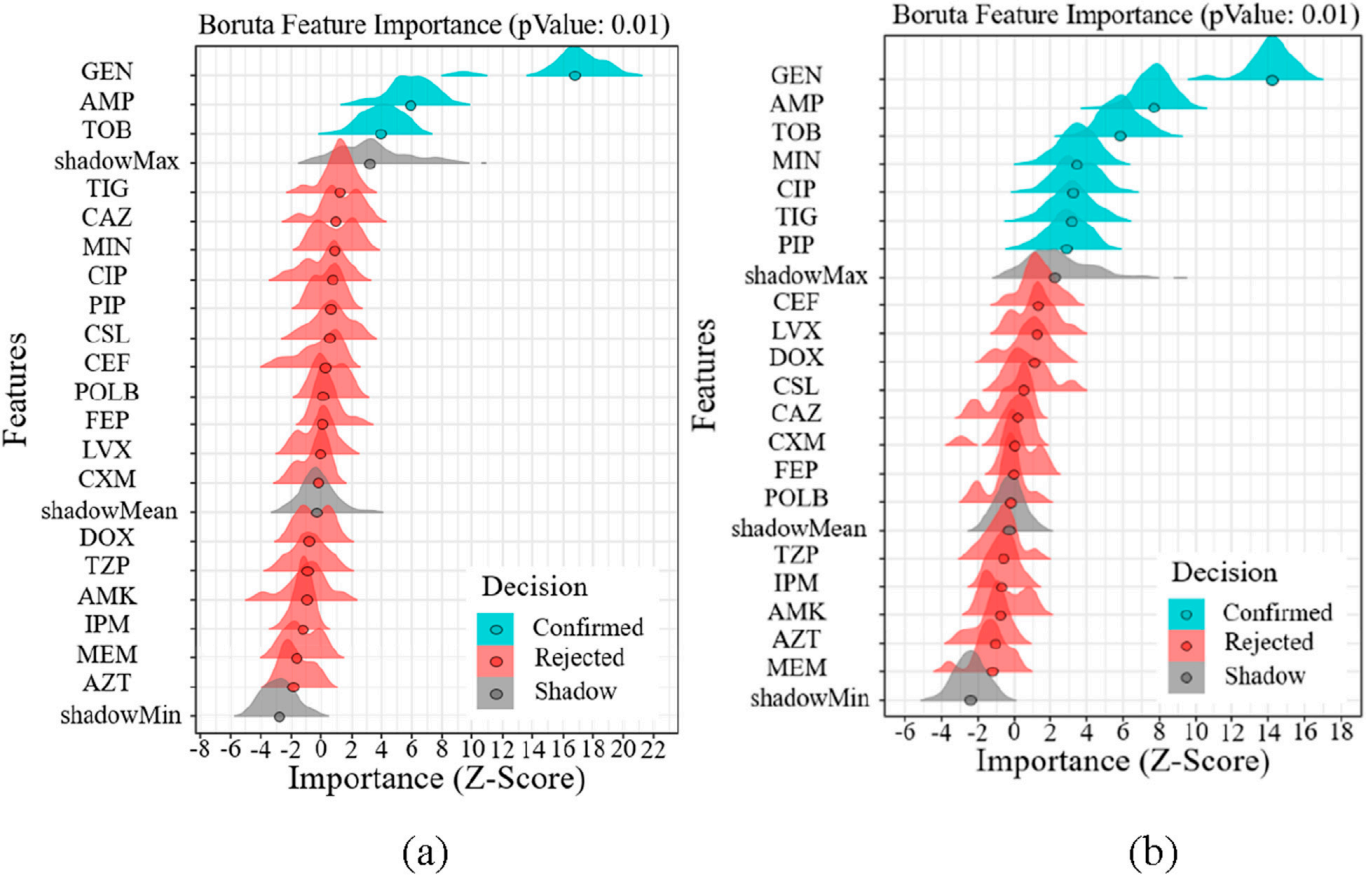
Feature importance analysis of Gram-positive bacteria. **(a)** Feature importance analysis of gender **(b)** Feature importance analysis of age.

Regarding the drug susceptibility analysis of Gram-negative bacteria, Figure 6 shows that the drug susceptibility of Gram-negative bacteria based on age and gender differs significantly from that of Gram-positive bacteria. Figure 6 illustrates how age increases drug susceptibility to Gram-negative bacteria and how the Boruta algorithm is important in filtering out more medication kinds. GEN, AMP, and TOB are the most gender-related medications, as shown in Figure 6a. The most age-related medications are GEN, AMP, TOB, MIN, and CIP, as shown in Figure 6b. GEN, AMP, and TOB are among the medications that are age and gender-specifically sensitive to Gram-negative bacteria.

### 3.5 Results of feature selection

**FIGURE 7:**
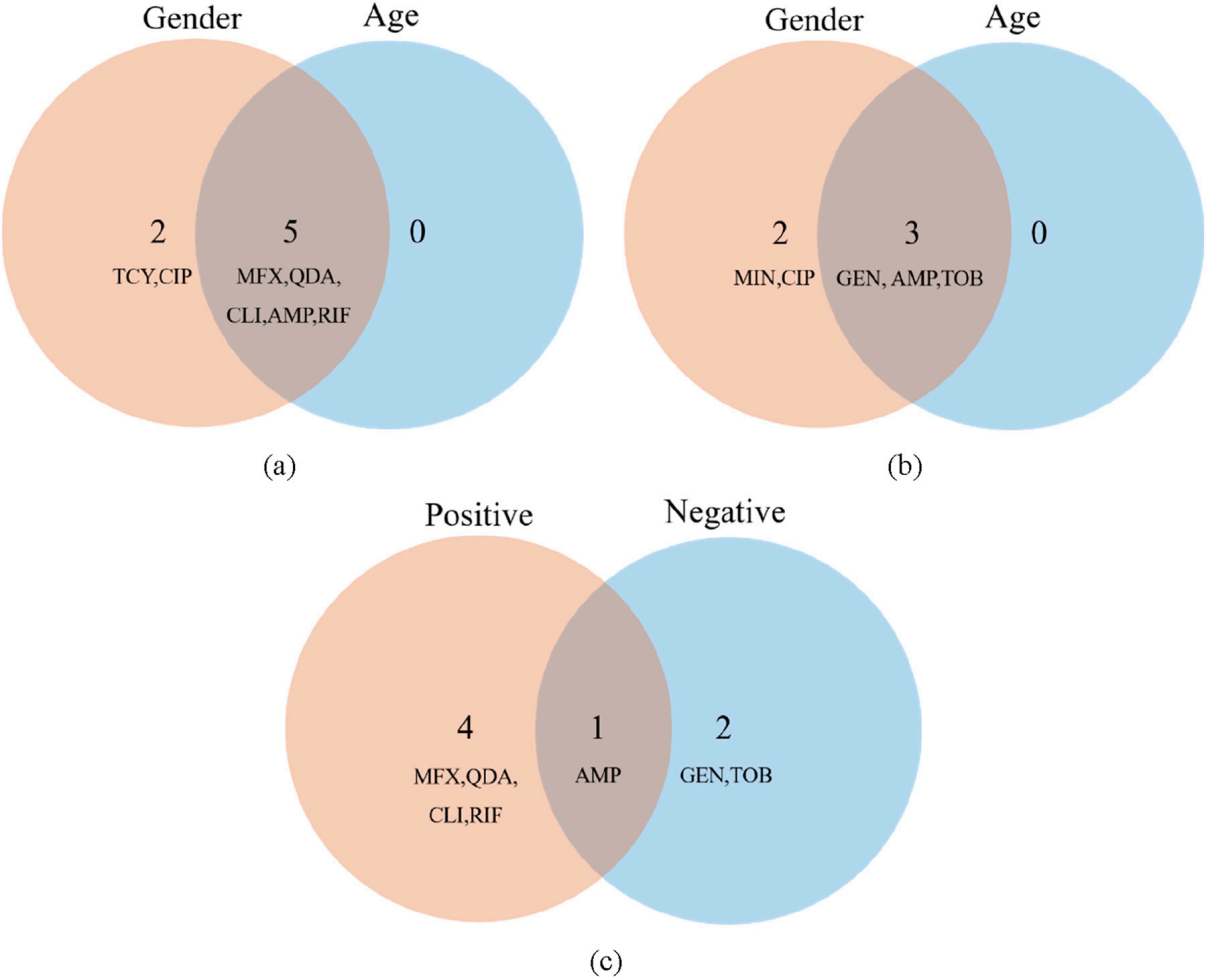
Features importance analysis. **(a)** Features importance analysis of gender **(b)** Features importance analysis of age **(c)** Features importance analysis of Gram-positive and Gram-negative bacteria.

In this work, we selected the salient features for various targets using the Boruta algorithm. The results of feature importance selection are listed in Figures 7a–c. Additionally, Figures 7a,b show significant characteristics associated with gender and age. The best characteristics of Gram-positive bacteria were found to be MFX, AMP, CLI, QDA, and RIF medications. The best characteristics of Gram-negative bacteria were found to be the medications GEN, AMP, and TOB. AMP is the only medication that is responsive to both Gram-positive and Gram-negative microorganisms based on age and gender (Figure 7c). At the same time, we reviewed the pertinent literature (Kuhn, 2013; Parvizi et al., 2013) and discovered that RIF and SXT were among the antibiotics that were prohibited from being added to bone cement due to the presence of Gram-positive microorganisms. Drug properties, chemical and physical compatibility, clinical efficacy, and safety are the primary causes. Sulfamethoxazole (SXT) has poor antibacterial efficacy against staphylococci, which is one of the primary reasons it shouldn’t be used to bone cements like PMMA (Beeching et al., 1986). The Hydroquinone structure of RIF, in particular, precludes its application in bone cement. This structure has the ability to catch free radicals, and as bone cement is being polymerized, a lot of free radicals will be created. The reaction between rifampicin and free radicals will prevent the polymerization of bone cement (Kuhn, 2013; Han et al., 2013). MFX, AMP, CLI, and QDA are the last Gram-positive bacterial antibiotics for drug susceptibility study, and RIF should be disregarded from the aforementioned characteristics. IPM and TIG are antibiotics that prevent bacteria from adding bone cement. The primary causes include issues with drug stability, restrictions on antibacterial activities, and so forth. Imipenem’s (IPM) chemical structure is typified by a double bond binding site in its second ring that is extremely susceptible to heat, benzoyl peroxide, and water. Because of the cement’s composition and the heat produced during polymerization, the imine structure is extremely vulnerable to hydrolysis and will lose its function if added to bone cement (Kuhn, 2013). Both the cumulative release of antibiotics and the antibacterial activity were minimal when imipenem was added to bone cement alone (Cerretani et al., 2002). Its pharmacological characteristics and the physicochemical characteristics of bone cement are the primary causes of the poor tigecycline elution in bone cement. Tigecycline is a lipid-soluble medication with weak water solubility (0.6–1.2 mg/mL at pH 7). In contrast, bone cement (like PMMA) has low elution efficiency because drug release is dependent on water penetration and drug dissolution. Due to its large molecular weight (585.6 Da), tigecycline may have trouble diffusing through bone cement’s pores. Consequently, it is not advised to combine tigecycline with bone cement to create antibiotic bone cement (Nichol et al., 2017). Since they are not recommended for incorporation into bone cement, they were not included in the study.

### 3.6 Drug sensitivity analysis

**FIGURE 8:**
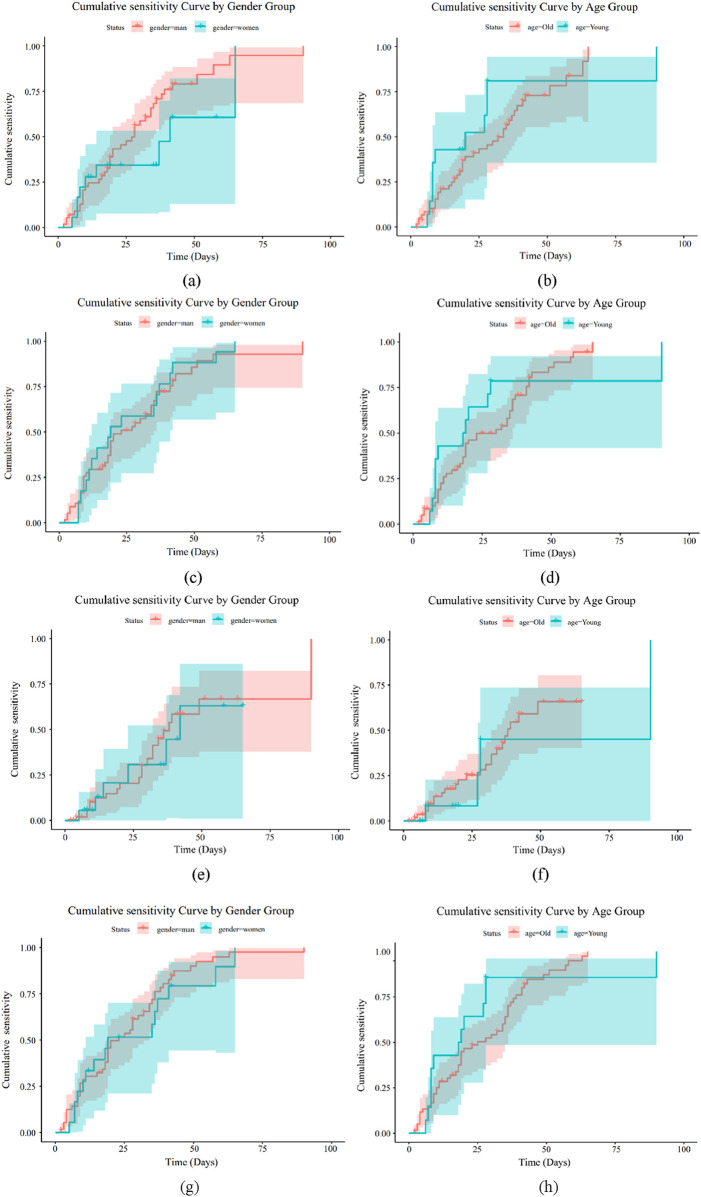
Drug sensitivity analysis of Gram-positive bacteria. **(a)** Drug MFX sensitivity analysis of gender **(b)** Drug MFX sensitivity analysis of age. **(c)** Drug AMP sensitivity analysis of gender **(d)** Drug AMP sensitivity analysis of age. **(e)** Drug CLI sensitivity analysis of gender **(f)** Drug CLI sensitivity analysis of age. **(g)** Drug QDA sensitivity analysis of gender **(h)** Drug QDA sensitivity analysis of age.

**FIGURE 9:**
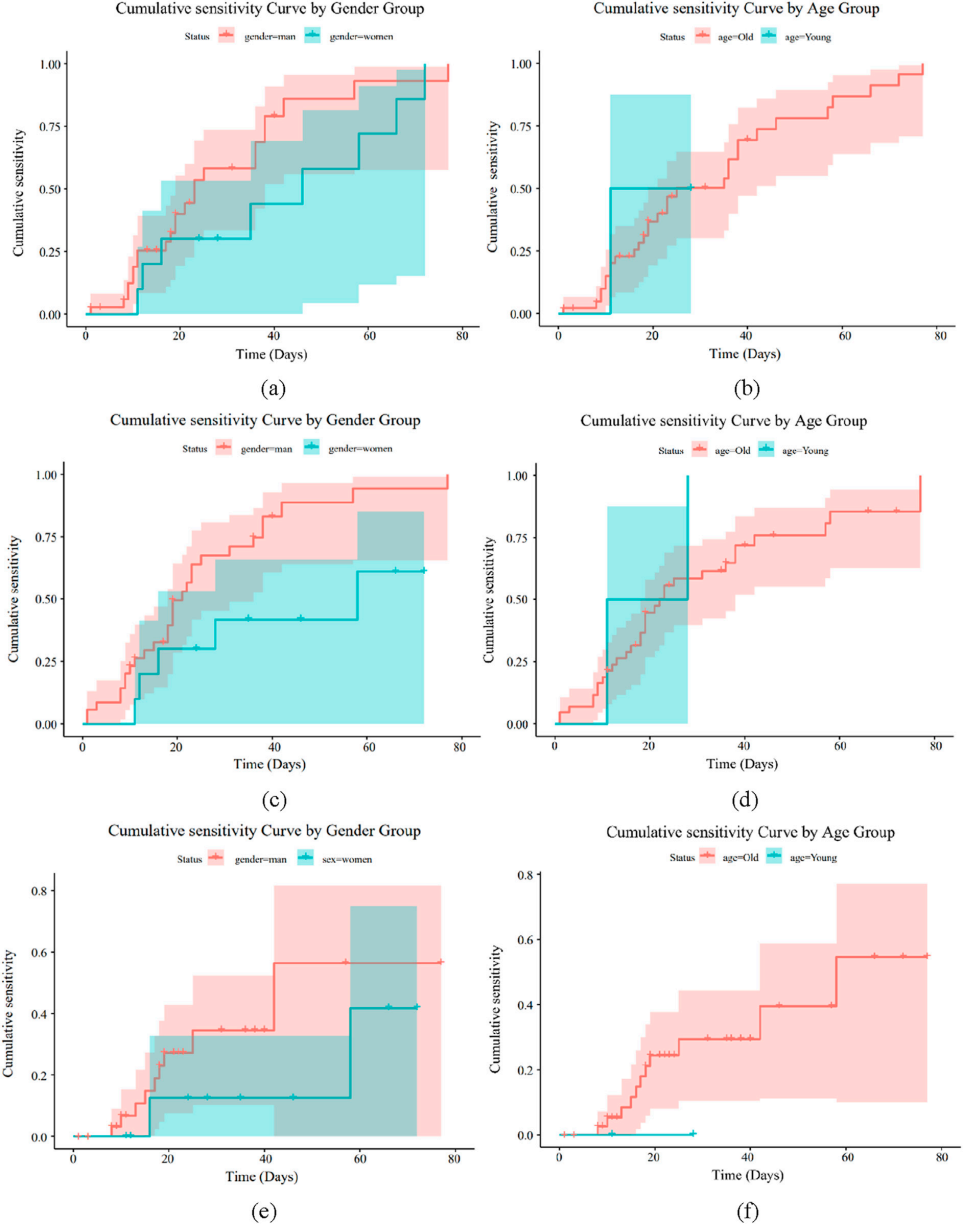
Drug sensitivity analysis of Gram-negative bacteria. **(a)** Drug GEN sensitivity analysis of gender **(b)** Drug GEN sensitivity analysis of age. **(c)** Drug TOB sensitivity analysis of gender **(d)** Drug TOB sensitivity analysis of age. **(e)** Drug AMP sensitivity analysis of gender **(f)** Drug AMP sensitivity analysis of age.

Using feature screening and antibacterial drug usage guidelines, the kinds of antibiotics that both Gram-positive and Gram-negative bacteria can add to bone cement were identified. We then looked into the cumulative sensitivity of antibiotics over time at various ages and genders, using the hospitalization period as the abscissa and the cumulative medication susceptibility as the ordinate. Figures 8, 9 display the cumulative sensitivity analysis curves of both Gram-positive and Gram-negative microorganisms with various antibiotics. The cumulative sensitivity analysis of the medication MFX is displayed in Figures 8a,b. Figure 8a shows that the antibiotic MFX peaks for women after 40 days in the hospital and for men after 60 days Figure 8b illustrates that the antibiotic MFX peaks at 60 days in hospital for patients over 50 and at 27 days in hospital for patients under 50. Overall, men and those over 50 are more susceptible to drug MFX. The antibiotic AMP peaks on the 55th day of hospitalization and does not appear to differ significantly across the sexes, as shown in Figure 8c. Figure 8d shows that the antibiotic AMP peaks at 60 days in the hospital for patients over 50 and at 27 days in the hospital for patients under 50. Overall, patients over 50 are more susceptible to the medication MFX. It is evident from Figure 8e that there is no discernible sex difference in antibiotic CLI, which peaks approximately 50 days following hospitalization. Figure 8f shows that the antibiotic CLI peaks about 50 days after hospitalization for patients over 50 and at roughly 25 days after hospitalization for patients under 50. Overall, people over 50 are more sensitive to medication CLI. Figure 8g shows that the antibiotic QDA peaks after roughly 60 days in the hospital and does not clearly differ between the sexes. The antibiotic QDA peaks at 60 days in the hospital for patients over 50 and at roughly 25 days in the hospital for patients under 25. This is evident from Figure 8h. Patients over 50 are generally more susceptible to QDA. To sum up, those over 50 are more susceptible to the effects of antibiotics for Gram-positive microorganisms. In conclusion, MFX, AMP, and QDA shown notable cumulative sensitivity to age and sex; hence, in Gram-positive bacteria, AMP and QDA were suggested for antibiotics coupled with bone cement.

Figures 9a–f illustrate how sensitive Gram-negative bacteria are to drugs. Figures 8a,b show the cumulative sensitivity curve of antibiotic GEN. Figure 9a makes the cumulative sensitivity of antibiotic GEN to men more obvious. The antibiotic GEN constantly affects persons over 50, but its effects on those under 50 gradually level out after 15 days in the hospital, according to Figure 9b, which depicts the age distribution. Men over 50 are generally more sensitive to the medication GEN. The cumulative sensitivity of antibiotic TOB to men is more evident and persisted during the hospital stay, as shown in Figure 9c. Regarding age, Figure 9d shows that the antibiotic TOB continuously affects the group over 50, whereas at 15 days of hospitalization, the effect on the group under 50 gradually stabilizes. Generally speaking, TOB is more sensitive to men over 50, much like GEN. Figure 9f illustrates that antibiotic AMP is nearly insensitive to individuals under 50 but extremely sensitive to the cumulative sensitivity of those over 50. Regarding age, Figure 9e shows that the male population is more affected by antibiotic AMP than the female population. Men over 50 are generally more responsive to antibiotics for Gram-negative bacteria. In conclusion, GEN and TOB demonstrated considerable cumulative sensitivity to both age and sex, while AMP has no cumulative sensitivity for women or individuals under 50. As a result, GEN and TOB were suggested for antibiotics coupled with bone cement in Gram-negative bacteria.

## 4 Discussion

DFU significantly lowers diabetic patients’ quality of life and is a major consequence of diabetes (Price, 2004). Diabetic patients are at risk for foot ulcers, necrosis, bacterial infections, and problems with lower limb blood circulation because of peripheral vascular disease. Non-traumatic lower limb amputation (LEA) is required for the majority of patients, which ultimately results in a high death rate (Cramb et al., 2021; Mader et al., 2019). Antibiotic-loaded bone cement has shown promising clinical results as a novel therapy for diabetic foot ulcers. PMMA bone cement is a type of biological substance that falls within the acrylic resin polymer category. It was initially applied in dentistry before progressively spreading to orthopedics (Bistolfi et al., 2011; Jaeblon, 2010). However, bone cement is vulnerable to bacterial adhesion and infection at the bone cement interface due to its lack of antibacterial qualities. DFU patients are susceptible to infection due to a generalized imbalance in their immunological response. Consequently, a certain level of antibacterial activity is provided by adding antibiotics to bone cement in order to treat DFU wounds (Bistolfi et al., 2019). In addition to promoting the release of various cytokines and causing the creation of fibrous membranes rich in blood vessels in the surrounding tissues, bone cement can hasten the healing of DFI wounds (Liu et al., 2019).

After Buchholz and Lodenkamper’s 1969 proposal to inject antibiotics into acrylic bone cement, which was primarily used to treat joint and bone infections, and its successful clinical outcomes, the practice became popular (Dai et al., 2023; Lachiewicz et al., 2020). The flow of anti-infective medicines alone is insufficient due to the restricted blood supply surrounding the affected tissue, and systemic antibiotic therapy does not reach those areas of inflammation, infection, or necrosis early. Then, huge quantities of topical antibiotics can be released, giving sick tissues and organs even more protection. PMMA bone cement that contains antibiotics is crucial in this situation. The use of bone cement containing antibiotics can lower the incidence of infection by roughly 50%, according to a meta-analysis research by Parvizi on the efficacy of PMMA bone cement in total hip arthroplasty (Parvizi et al., 2008). The local ulcerated infection in diabetic foot can also be effectively treated with bone cement that contains antibiotics. As a result, when choosing antibiotics, we need to take into account: i) The polymerization process of bone cement is stable in the face of heat and chemical reactions; ii) It has minimal impact on the hardening and strength of bone cement; iii) It has good release from solidified bone cement; iv) It has a broad antibacterial spectrum and good antibacterial effect at low concentrations; v) The rate of bacterial resistance is low; vi) It has low protein binding; vii) It has a low allergy rate; and viii) It has good cell penetration.

DFU pathogens often exist in biofilms. A bacterial biofilm is a kind of aggregated membrane-like structure formed by bacteria adhering to the surface of an object. Approximately 80% of the bacterial biofilm is non-crystalline layer, while only 20% consists of bacteria. Biofilms can effectively protect microorganisms, preventing them from being recognized and minimizing their metabolic rate. Antibacterial drugs have great difficulty penetrating the biofilm. This means that very high drug concentrations, even 1000 times the normal concentration, are required to affect the bacteria within the biofilm. Such a concentration is difficult to achieve when using the medication throughout the entire body. Antibiotic bone cement has certain advantages in eliminating bacterial biofilms. i) High-concentration local release of antibiotics: After the antibiotic bone cement is implanted at the infected site, it will continuously and slowly release high concentrations of antibiotics into the local environment. This local drug concentration is much higher than that achievable through systemic administration, enabling more effective penetration and destruction of biofilms and killing the bacteria encapsulated within them. ii) Provide mechanical support and fill the dead space: In addition to anti-infection, antibiotic bone cement can also play a mechanical stabilizing role and fill the bone defect cavities caused by debridement, reducing the space where bacteria can hide. iii) Inhibition of biofilm formation: Local high concentrations of antibiotics can effectively prevent bacteria from adhering and aggregating on the surface of implants, thereby preventing the initial formation and further development of biofilms. Of course, for stubborn bacterial biofilms, we also need to perform an effective and thorough debridement procedure before implanting the antibiotic bone cement, and replace the antibiotic bone cement multiple times. The local use of antibiotics aims to quickly achieve an effective drug concentration, avoiding the adverse reactions caused by long-term systemic use of antibiotics. However, it still has potential local cytotoxicity and risks of systemic absorption. The research found that these localized release concentrations exceeded the sensitivity limit of the pathogens, allowing them to be cleared before the formation of bacterial biofilms (Markakis et al., 2018).

Of the 102 samples we examined, the majority (about 83.31%) had a single bacterial infection. Approximately 75.63% of them were Gram-positive bacteria, with *Staphylococcus aureus* being the most prevalent Gram-positive bacterium. There was no discernible preponderance of Gram-negative bacteria, with *Proteus mirabilis*, *Escherichia coli*, and *Klebsiella pneumoniae* making up roughly 7%–8% of the total. Iranian researcher Shahrokh who did a meta-analysis discovered that *Staphylococcus aureus* was the predominant strain (24.29%). *Escherichia coli* made up the largest percentage of Gram-negative bacteria (17.19%), with *Pseudomonas aeruginosa* coming in second (7.54%) (Shahrokh et al., 2022). In a meta-analysis of the bacterial microbiota of diabetic foot wounds across the globe, Hawkins, an American, discovered that *Staphylococcus aureus* was the most prevalent bacterium in Europe, America, and Asia. Geographical differences in Asia caused the bacterial composition of Gram-negative bacteria to differ, but *Pseudomonas aeruginosa* to be relatively more prevalent (Hawkins et al., 2022). In a multicenter study on the bacterial microbiota of diabetic foot wounds in Latin America, the results revealed that the proportion of Gram-positive bacteria was relatively high, with *Staphylococcus aureus* being the most prevalent bacteria (Carro et al., 2022). Ethiopian researchers examined the microbiota of African diabetic foot patients and found that *Staphylococcus aureus* was the predominant bacterium (25.19%) and that Gram-negative bacteria made up a comparatively large percentage (66.7%). *Escherichia coli* (16.53%) and *pseudomonas* (18.89%) made up a comparatively large percentage of the Gram-negative bacteria (Atlaw et al., 2022). While *Enterobacter* and *Pseudomonas* are reasonably frequent Gram-negative bacteria, *Staphylococcus aureus* is unquestionably the most prevalent species in diabetic foot wounds globally, according to these studies, which closely resemble our findings. The findings of this study give us a solid foundation on which to choose antibiotics more wisely and prevent the emergence of drug resistance.

The selection of antibiotics is especially crucial when they are added to bone cement to treat diabetic foot wounds, so we examined the drug susceptibility test results of these bacterial samples. Using the Boruta algorithm prediction model, we first screened the antibiotics and then performed sensitivity analysis based on age and gender factors. We discovered that the best antibiotics for Gram-positive bacteria were moxifloxacin (MFX), ampicillin (AMP), and Quinupristin-dalfopristin (QDA), while the best agents for Gram-negative bacteria were gentamicin (GEN) and tobramycin (TOB). A fourth-generation quinolone antimicrobial agent, moxifloxacin (MFX) is mostly used to treat respiratory tract infections. It has a broad-spectrum antibacterial impact, strong antibacterial activity, good drug resistance, low phototoxicity, and high absorption (Hoogkamp-Korstanje and Roelofs-Willemse, 2000; Blondeau, 1999; Boswell et al., 1999). Bacterial DNA gyrase and DNA topoisomerase are inhibited by moxifloxacin, which can bind topoisomerase Ⅱ and topoisomerase Ⅳ to destroy bacterial DNA and accomplish sterilization (Boswell et al., 1999; Chu and Fernandes, 1989; Schneider et al., 1990). Ampicillin is a broad-spectrum β-lactam antibiotic that is semisynthetic. It exerts a bactericidal action by blocking the synthesis of bacterial cell wall. It can attach to penicillin-binding proteins (PBPs) on the bacterial cell membrane and interfere with the formation of peptidoglycan in the cell wall, resulting to the defect of the bacterial cell wall and swelling and lysis of the bacterial body. Both Gram-positive and Gram-negative bacteria are susceptible to its antibacterial properties (Brunton et al., 2018). According to the data we have collected, the rate of *Staphylococcus aureus* resistance to AMP among patients with diabetic foot is 17%. Although AMP has a general resistance to *S. aureus*, it shows good sensitivity to *Enterococcus faecalis*. Taking all factors into consideration, AMP can be preliminarily and empirically used. Once the patient’s drug sensitivity results are available, specific sensitive antibiotics for the individual can be selected for treatment. The sole streptocin medication is Quinupristin-dalfopristin (QDA), which is a 30/70 mixture of two distinct forms of natural insoluble prist-inamycin derivatives. By attaching two distinct pharmacological components to various locations inside the bacterial ribosome’s 50s subunit, it prevents the synthesis of proteins (Barrière et al., 1998). Against Gram-positive cocci, QDA had potent antibacterial action, and the antibacterial impact was unaffected by the findings of resistance to other antimicrobial drugs (Bouanchaud, 1997). We obtained the effective recommendations for QDA through data processing using predictive models. However, due to its cost, availability, and side-effect profile, the selection of this drug requires comprehensive consideration by clinical doctors. By analyzing the drug sensitivity data of the bacterial flora in diabetic foot infections, the resistance rates of MFX, AMP and QDA were 29%, 20% and 13% respectively. All three antibiotics can be added to bone cement and are effective against Gram-positive bacteria. One of the primary antibiotics used in clinical practice to treat a variety of Gram-negative bacterial infections is gentamicin (GEN). It is commonly utilized and has a high release in the first 24 h and a continuous low release of antibiotics for up to 50 days in bone cement (Bitsch et al., 2015). One of the first antibiotics to be utilized in bone cement was gentamicin (GEN), which was first applied to infections from mechanical joint replacements (Dai et al., 2023). The primary purpose of tobramycin (TOB), an aminoglycoside antibiotic with a broad antibacterial range, is to fend off Gram-negative bacterial infections, particularly those caused by *Pseudomonas aeruginosa*. It may be removed easily and has good thermal stability in bone cement (Scott et al., 1999). The resistance rates of GEN and TOB against Gram-negative bacteria were 31% and 22%, respectively. Relevant studies have proved that GEN, TOB, MFX, AMP and QDA all have good compatibility with bone cement, and their stability in bone cement is also relatively good (Kuhn, 2013; Parvizi et al., 2013). Studies have shown that when 0.5 g of antibiotic is added to 40 g of bone cement, the total release amounts of GEN, TOB and AMP over 10 days are 2900 μg, 2100 μg and 800 μg respectively. Containing moxifloxacin bone cement, the drug release was relatively rapid 14 days ago, and then it gradually became more stable (Wahlig, 1987). There are relatively few studies on the dynamics of bone cement related to QDA, and further research is needed to better understand its characteristics.

With the widespread use of penicillin, bacteria that were resistant to it emerged. The first methicillin-resistant *Staphylococcus aureus* (MRSA) strain was identified as early as 1961. The core resistance of MRSA is resistance to β-lactam antibiotics. The detection rate of MRSA in our hospital is 18.75%.For the treatment of MRSA, the first step is to perform a thorough surgical debridement, followed by the administration of vancomycin mixed with bone cement for treatment. Adding vancomycin to bone cement can have a very good therapeutic effect on various different MRSA strains. For Gram-negative bacteria with strong drug resistance, we use meropenem and add it to the bone cement for treatment. Of course, during the process of overusing antibiotics to control infections, one must also be cautious about the occurrence of fungal infections in diabetic foot wounds.

An excellent option for treating diabetic foot wound infections is to empirically implant these antibiotics into bone cement. Naturally, we should also take into account the stability, release capacity, and physicochemical characteristics of antibiotics in bone cement. Before applying antimicrobial bone cement to the diabetic foot wound, adequate debridement surgery is required. The optimal dosage of antibiotics is still unknown. Studies have shown that high-dose antibiotic bone cement (with more than 2 g of antibiotics in 40 g of bone cement) and low-dose antibiotic bone cement (with less than 2 g of antibiotics in 40 g of bone cement) can be used for prevention (Wall et al., 2021). The most important rule when manually adding antibiotics to bone cement is that no more than 4 g of antibiotics should be added to 40 g of bone cement. We advise changing the antibiotic cement at least 3 weeks after the peak antibiotic release in the cement. Occasionally, repeated debridement treatments may be necessary to replace the antibiotic cement and produce satisfactory outcomes.

Our study was a single-center investigation, the sample size was small, and the findings have certain limitations. Superficial infections of DFI are mainly caused by aerobic Gram-positive cocci, mostly by single-bacteria infection, and only a few are mixed infections. Deep infections are usually caused by multiple pathogenic bacteria, with Gram-negative bacteria being the main type, followed by Gram-positive bacteria and anaerobic bacteria. We do not routinely culture anaerobic bacteria and special flora. Further culture and identification are only conducted under highly suspicious or special circumstances. This limits the comprehensive identification of bacteria. For cases of multiple bacterial infections, we usually only analyze two of the main bacteria. If more than two species are cultivated, we might consider the specimens to be contaminated and conduct another test. This could also limit our analysis of multiple bacterial infections. The way we cultivate is rather monotonous and lacks diversity,it may miss non-culturable organisms. We will integrating next-generation sequencing for microbiome profiling to capture uncultivable species.

In summary, the use of antibiotic-loaded bone cement is a relatively novel and successful treatment for DFI, and the selection of antibiotics is a critical component of this approach. Our study’s findings should aid medical professionals in the management of DFU. Next, it is necessary for us to conduct relevant experiments to compare the effects of systemic antibiotics and antibiotic-loaded bone cement in the treatment of diabetic foot ulcers.

## 5 Conclusion

According to this study, age was a significant factor in the cumulative sensitivity of Gram-positive bacteria to antibiotics added with bone cement, while age and gender, particularly age, were significant factors in the cumulative sensitivity of Gram-negative bacteria to antibiotics added with bone cement. In the bacterial microbiota of DFU wounds, Gram-positive bacteria accounted for a relatively high proportion, and the main species was *Staphylococcus ureus*. Based on the aforementioned findings, it is advised that the DFU population who requires antibiotic-loaded bone cement treatment be treated according to age, with GEN and TOB being recommended for Gram-negative bacteria and MFX, AMP, and QDA for Gram-positive bacteria. Of course, these medications are recommended for the initial use of antibiotic bone cement. However, the precise treatment for patients still requires a rational selection based on the results of bacterial culture and drug sensitivity tests. Furthermore, our study’s findings have certain limitations, and we anticipate larger sample sizes and multi-center prospective randomized controlled trials to allow for additional discussion. In summary, diabetic foot ulcer infection can be effectively treated using antibiotic-loaded bone cement, a relatively novel technique.

## Data Availability

The raw data supporting the conclusions of this article will be made available by the authors, without undue reservation.

## Glossary

DFU: diabetic foot ulcer
DFO: Diabetic foot osteomyelitis
VSD: vacuum sealing drainage
DFI: diabetic foot infections
Sho: *Staphylococcus* hominis
Efaa: *Enterococcus faecalis*
Spy: *Streptococcus* pyogenes
Cje: Corynebacterium jeikeium
Sau: *Staphylococcus aureus*
Ost: Oral Streptococci
Slu: *Staphylococcus* lugdunensis
Sha: *Staphylococcus haemolyticus*
Efai: *Enterococcus* faecium
Sdy: *Streptococcus* dysgalactiae
Cst: Corynebacterium striatum
Sag: *Streptococcus* agalactiae
Sco: *Streptococcus* constellatus
Sin: *Staphylococcus* intermedius
Cko: *Citrobacter* Koseri
Pre: *Providencia rettgeri*
Mmo: *Morganella morganii*
Pvu: *Proteus.vulgaris*
Pmi: *Proteus mirabilis*
Smal: *Stenotrophomonas maltophilia*
Pst: *Providencia stuartii*
Pae: *Pseudomonas aeruginosa*
Sly: *Soil lysobacterium*
Ecl: *Enterobacter cloacae*
Smar: *Serratia marcescens*
Eco: *Escherichia coli*
Kpn: *Klebsiella pneumoniae*
Kae: *Klebsiella aerogenes*
Ppu: *Pseudomonas putida*
Cfr: *Citrobacter freundii*
Eho: *Enterobacter hormaechei*
Sep: *Staphylococcus epidermidis*
